# Predictors of response to bevacizumab monotherapy in polypoidal choroidal vasculopathy: a 12-month retrospective study

**DOI:** 10.1186/s40942-025-00795-x

**Published:** 2026-01-07

**Authors:** Chinnapat Montrisuksirikun, Supredee Pongrujikorn, Nuttawut Rodanant, Somanus Thoongsuwan, Supalert Prakhunhungsit, Nida Wongchaisuwat, Nopasak Phasukkijwatana

**Affiliations:** Department of Ophthalmology, Faculty of Medicine Siriraj Hospital, Mahidol University, Bangkok, 10700 Thailand

**Keywords:** Polypoidal choroidal vasculopathy (PCV), Bevacizumab, Optical coherence tomography (OCT), Biomarkers

## Abstract

**Background:**

To identify baseline predictors of anatomical response after intravitreal bevacizumab in polypoidal choroidal vasculopathy (PCV) and to evaluate anatomical and visual outcomes over 12 months.

**Methods:**

This retrospective study included 81 eyes with PCV treated with three consecutive monthly intravitreal bevacizumab injections. Patients were classified as good or poor responders based on optical coherence tomography (OCT) findings at month 3. Baseline demographic, clinical, and imaging parameters were compared between groups, and predictors of poor response were identified using multivariable regression. Visual acuity (VA), central retinal thickness (CRT), subretinal fluid (SRF), and pigment epithelial detachment (PED) height were analyzed within and between groups through month 12.

**Results:**

Forty eyes (49.4%) were poor responders and 41 (50.6%) were good responders. Greater baseline SRF height and thinner SFCT were independent predictors of poor response (RR per 100 μm: 1.27 and 0.71 respectively; AUC = 0.74). Good responders showed significant VA improvement (0.64 ± 0.08 vs. 0.48 ± 0.06 logMAR, *p* < 0.001) and CRT reduction (419.5 ± 24.3 vs. 285.5 ± 14.1 μm, *p* < 0.001) at month 3, and these were maintained at month 12. Among good responders, 75% continued bevacizumab injections (non-switch group) and sustained favorable outcomes. In contrast, 86% of poor responders switched to other medications (switch group), achieving significant VA gain, CRT reduction, and further SRF with PED reduction by month 12. The non-switch group required fewer injections (5.8 ± 2.5 vs. 7.8 ± 3.0 injections; *p* < 0.001) and achieved longer treatment intervals (11.7 ± 9.0 vs. 7.8 ± 3.0 weeks; *p* = 0.046) than the switch group.

**Conclusions:**

Approximately half of PCV eyes demonstrated a favorable response to bevacizumab monotherapy. Baseline SRF height and SFCT were significant predictors of treatment response. Early assessment after three monthly bevacizumab injections allowed for the differentiation between relatively mild and a more severe disease requiring more frequent and sustained anti-VEGF injections. Switching of anti-VEGFs was associated with improved outcomes in poor responders.

**Supplementary Information:**

The online version contains supplementary material available at 10.1186/s40942-025-00795-x.

## Introduction

Polypoidal choroidal vasculopathy (PCV) is currently classified as a subtype of neovascular age-related macular degeneration (nAMD), which is one of the leading causes of irreversible vision loss among the elderly worldwide. PCV is characterized by abnormal vascular formation in the choroid leading to serosanguinous maculopathy. The condition is recognized for its clinical and imaging characteristics as described in the EVEREST study [1]. 

There are two treatment approaches for PCV, intravitreal anti-VEGF monotherapy, and a combination of anti-VEGF and photodynamic therapy (PDT). Previous studies demonstrated favorable outcomes after anti-VEGF monotherapy in PCV [2, 3]. Although bevacizumab is not FDA-approved for PCV, studies have reported outcomes comparable to those of the FDA-approved ranibizumab for PCV [4–6]. Aflibercept may be more effective than bevacizumab and ranibizumab for PCV [2, 7]. Nevertheless, bevacizumab remains a first-line agent for nAMD and PCV in many healthcare systems because of its cost-effectiveness [8–10]. In Thailand, bevacizumab is the only anti-VEGF included in the National Drug List of Essential Medicines for the treatment of macular diseases [10]. 

Despite its widespread use, there is considerable variability in the patient’s response to bevacizumab, with some individuals experiencing substantial visual gains, while others showing minimal improvement or even continued disease progression. Identifying factors that contribute to this variability could facilitate more personalized treatment approaches, which could involve early intervention with other anti-VEGF agents or combination therapies [11]. 

The research on ranibizumab and aflibercept has identified several prognostic factors associated with treatment response, including central subfoveal thickness (CRT), subfoveal choroidal thickness (SFCT), pachyvessels, choroidal hyperpermeability (CVH), and polyp area [12–16]. However, the results of predictive factors for using bevacizumab in PCV are still limited. This study aims to identify predictive factors for patients who exhibit poor response to bevacizumab and to demonstrate the 1-year efficacy of bevacizumab monotherapy for PCV.

## Methods

This study is a single-center retrospective cohort study. The electronic medical records of patients diagnosed with polypoidal choroidal vasculopathy (PCV) at Siriraj Hospital were reviewed. The study was conducted consecutively in accordance with ethical guidelines for retrospective data collection and was approved by the Institutional Review Board and Ethics Committee of Siriraj Hospital, Mahidol University (COA Si 629/2023). It adhered to the tenets of the Declaration of Helsinki. This study was retrospectively registered in Thai Clinical Trial Registry on 22 September 2024 (TCTR20241227005; https://www.thaiclinicaltrials.org).

### Inclusion and exclusion criteria

Patients diagnosed with PCV according to the EVEREST diagnostic criteria [1] who met the following inclusion criteria were enrolled in the study. The additional inclusion criteria were: (1) age 18 years or older; (2) Pinhole visual acuity (VA) of 20/32 or worse, with the presence of serosanguinous maculopathy involving the macula (within a 5500 μm diameter centered on the fovea); (3) Being treated with a loading phase of 3 monthly injections of intravitreal bevacizumab (1.25 mg/0.05 ml); and (4) availability of baseline imaging, including optical coherence tomography (OCT), fundus photography, and indocyanine green angiography (ICGA).

Exclusion criteria included patients who: (1) had previous treatments with intravitreal anti-VEGF, verteporfin photodynamic therapy (PDT), focal laser photocoagulation, or pneumatic displacement of subretinal blood; (2) presented with massive subretinal hemorrhage that obscured ICGA interpretation; (3) had a history of pathological myopia, retinal detachment, macular hole, or uncontrolled glaucoma; (4) had coexisting conditions that could cause macular edema, such as diabetic macular edema, posterior uveitis, or retinal vein occlusion; and (5) had undergone intraocular surgery (except uncomplicated cataract extraction with intraocular lens implantation) within 60 days before the first visit.

### Data collection

Baseline characteristics were collected at the initial clinic visit, which included a pertinent medical history, visual acuity (VA), eye examination, and retinal imaging characteristics. During months 3 and 12, VA, ocular examination, OCT parameters, anti-VEGF medication, treatment regimen, and injection interval at each visit were collected. In patients who underwent anti-VEGF switching, the reasons for switching were recorded. These reasons included persistent IRF or SRF, worsening IRF or SRF, new submacular hemorrhage, or failure to extend treatment interval.

### Retinal imaging

Retinal imaging assessments included color fundus photography (Non-Mydriatic AFC-330; NIDEK Co., Ltd., Aichi, Japan); spectral-domain OCT (SD-OCT) with enhanced depth imaging (EDI-OCT) (Spectralis, Heidelberg Engineering, Heidelberg, Germany); and simultaneous fluorescein angiography (FA) and indocyanine green angiography (ICGA) (Spectralis, Heidelberg Engineering, Heidelberg, Germany). The specific parameters evaluated included:

- Fundus photography parameters at baseline: presence of orange nodules, subretinal hemorrhage, and hard exudates.
- OCT parameters: central retinal thickness (CRT), subfoveal choroidal thickness (SFCT), presence of intraretinal fluid (IRF) and subretinal fluid (SRF), maximum SRF height, maximum pigment epithelial detachment (PED) height at baseline, months 3 and 12, and presence of a hyperreflective ring in PED at baseline.
- FA and ICGA parameters at baseline: morphology of polyps, location and quadrant of polyps, presence of pulsatile polyps, largest diameters of polyps, leakage from polyps, presence of branching vascular network (BVN), leakage from BVN, area of BVN, area of polyp and BVN, and greatest linear dimension (GLD) of the lesion, and choroidal vascular hyperpermeability (CVH).

CRT and automated retinal layer segmentation were obtained using the device’s built in automated software. All B-scans were then reviewed and segmentation errors were manually corrected. Additional parameters were measured manually with the device’s caliper tools. Retinal images were independently reviewed by two retina specialists (CM, NP). In cases of discrepancy between the two readings, a joint review session was conducted to achieve consensus.

### Patient response classification

Patients were classified based on their anatomical response to treatment following three consecutive monthly intravitreal bevacizumab injections into good response and poor response groups [17]. Good response group included patients demonstrated complete resolution of SRF, IRF, intraretinal cysts (IRC), or a achieved a reduction in CRT of more than 75% from baseline.

Poor response group included patients who exhibited less than a 75% reduction in CRT from baseline, developed new SRF or IRF, or showed unchanged or increased CRT, SRF, IRF, and/or PED compared with baseline.

Following the three-month loading phase, subsequent treatment regimens and durations varied at the discretion of the treating physician. Management options included a treat-and-extend regimen, pro re nata (PRN) dosing, medication switching, rescue photodynamic therapy (PDT), or treatment deferral.

### Statistical analysis

Continuous variables were presented as mean ± standard deviation (SD) or as median with interquartile range (IQR), and comparison between groups were performed using the unpaired t-test. Categorical variables were presented as percentages and compared using the Chi-square test. Longitudinal changes across repeated measures, including datasets with missing values, were analyzed using generalized estimating equations (GEE) assuming that missing data were missing at random. Univariable and multivariable analyses were conducted using modified Poisson regression, and results were reported as relative risks (RR) with 95% confidence intervals (CI). A p-value of less than 0.05 was considered statistically significant.

## Results

A total of 81 eyes of 81 patients were included in the study. After three monthly intravitreal bevacizumab injections, 40 eyes (49.4%) were classified as poor responders and 41 eyes (50.6%) as good responders. Baseline demographic, clinical and imaging characteristics were compared between the two groups, and regression analyses were performed to identify predictors of poor response.

On univariable regression analysis, six factors were significantly associated with poor response: older age (RR = 1.03; 95%CI 1.01–1.05, *p* = 0.005), hypertension (RR = 2.13; 95%CI 1.29–3.50, *p* = 0.003), higher CRT (RR = 1.001; 95%CI 1.0005–1.002, *p* = 0.003), lower SFCT (RR = 0.996; 95% CI 993-0.999, *p* = 0.042), presence of IRF (RR = 1.54; 95%CI 1.01–2.34, *p* = 0.044), and greater maximal SRF height (RR = 1.00; 95%CI 1.0003–1.003, *p* = 0.014 (Table 1).

**Table 1:** Comparison of baseline clinical characteristics between poor-responder and good-responder group

Clinical characteristic	Poor responder		Good responder		*P*-value	RR (95% CI)	
	(*n* = 40)		(*n* = 41)				
**Patient characteristic**							
Age (years), mean (SD)	68.3	(7.0)	62.3	(9.6)	**0.005**	1.03	(1.01–1.05)
Male sex	24	(60%)	19	(46.3%)	0.231	1.33	(0.84–2.10)
Diabetes mellitus	13	(32.5%)	8	(19.5%)	0.152	1.38	(0.89–2.13)
Hypertension	27	(67.5%)	13	(32%)	**0.003**	2.13	(1.29–3.50)
**Ocular examination**							
LogMAR VA, mean (SD)	0.69	(0.47)	0.63	(0.51)	0.642	1.10	(0.74–1.65)
IOP (mmHg), mean (SD)	13.6	(3.6)	13.4	(3.0)	0.784	1.01	(0.94–1.09)
Lens status							
Phakic	25	(62.5%)	32	(78.0%)		Reference	
Pseudophakic	15	(37.5%)	9	(22.0%)	0.104	1.43	(0.93–2.18)
**Fundus photo**							
Orange nodule	19	(47.5%)	21	(51.2%)	0.738	0.93	(0.60–1.44)
Subretinal hemorrhage	30	(75.0%)	22	(53.6%)	0.078	0.68	(0.45–1.04)
Exudate	12	(30.0%)	16	(39.0%)	0.41	0.81	(0.49–1.33)
**OCT**							
CST (µm), mean (SD)	519.6	(243.9)	419.5	(157.7)	**0.003**	1.001	(1.0005–1.002)
SFCT (µm), mean (SD)	214.5	(57.2)	248.7	(77.7)	**0.042**	0.996	(0.993–0.999)
Presence of IRF	14	(35.0%)	7	(17.1%)	**0.044**	1.54	(1.01–2.34)
Presence of SRF	39	(97.5%)	36	(87.8%)	0.216	3.12	(0.51–18.92)
Maximal SRF height (µm), mean (SD)	251.7	(129.8)	192.6	(116.5)	**0.014**	1.000	(1.0003–1.003)
Maximal PED height (µm), mean (SD)	348.4	(202.2)	353.4	(173.0)	0.91	0.99	(0.99-1.00)
Presence of hyperreflective ring in PED	18	(45.0%)	17	(41.5%)	0.75	1.07	(0.69–1.67)
**ICGA**							
Polyp							
Morphology of polyps							
Cluster morphology	25	(62.5%)	18	(43.9%)	0.105	1.47	(0.92–2.35)
Single morphology	11	(27.5%)	17	(41.5%)			
String morphology	4	(10.0%)	6	(14.6%)			
Location of polyps							
Subfoveal	10	(25.0%)	11	(26.8%)		Reference	
Juxtafoveal	7	(17.5%)	6	(14.6%)	0.721	1.13	(0.58–2.22)
Non-peripapillary extrafoveal	18	(45.0%)	17	(41.5%)	0.785	1.08	(0.62–1.88)
Peripapillary extrafoveal	5	(12.5%)	7	(17.1%)	0.745	0.88	(0.39–1.96)
Presence of pulsatile polyps	12	(30.0%)	17	(41.5%)	0.303	0.77	(0.47–1.27)
Largest polyp diameter (µm), mean (SD)	342.8	(178.1)	363.7	(157.0)	0.543	0.99	(0.99-1.00)
Presence of leakage of polyps	17	(42.5%)	19	(46.3%)	0.729	0.92	(0.59–1.45)
BVN							
Presence of BVN	39	(97.5%)	39	(95.1%)	0.623	1.50	(0.30–7.55)
Leakage of BVN	18	(45.0%)	14	(34.1%)	0.31	1.25	(0.81–1.93)
BVN area (mm^2^), median (IQR)	3.49	(1.72-0.5.96)	3.95	(2.13–7.40)	0.251	0.97	(0.91–1.02)
Polyps and BVN							
Total lesion area (mm^2^), median (IQR)	3.77	(2.27–6.38)	4.35	(2.44–7.60)	0.416	0.98	0.93–1.03
GLD of the lesions (µm), mean (SD)	3311.6	(1386.1)	3551.9	(1544.2)	0.477	0.99	(0.99-1.00)
Presence of CVH	27	(67.5%)	31	(75.6%)	0.400	0.82	(0.52–1.29)

VA-visual acuity, IOP-intraocular pressure, CST-central subfield thickness, SFCT-subfoveal choroidal thickness, IRF-intraretinal fluid, SRF-subretinal fluid, PED-pigment epithelial detachment, BVN-branching vascular network, GLD-greatest linear diameter, CVH-choroidal vascular hyperpermeability

On multivariable regression analysis, two factors remained independent predictors of poor response: greater maximal SRF height per 100 μm increment (RR = 1.27; 95%CI 1.05–1.54, *p* = 0.012) and lower SFCT per 100 μm increment (RR = 0.71; 95%CI 0.50–0.99, *p* = 0.049) (Table 2). In the multivariable logistic regression model including all significant baseline predictors, the ROC analysis showed an AUC of 0.79. While the model incorporating only SRF height and SFCT achieved an AUC of 0.74.

**Table 2:** Multivariable analysis for poor-responder group

Factors^†^	Relative risk (RR)	95%-CI	*P*-value	
Age (10 years)^††^	1.20	0.90–1.60	0.207	
Hypertension	1.51	0.86–2.64	0.149	
SRF height (100 μm)^††^	1.27	1.05–1.54	**0.012**	
SFCT (100 μm)^††^	0.71	0.50–0.99	**0.049**	
Presence of IRF	1.27	0.82–1.98	0.284	

SRF-subretinal fluid, SFCT-subfoveal choroidal thickness, IRF-intraretinal fluid

† Central retinal thickness was related to SRF height and was excluded from the multivariable analysis

†† The RR is calculated for an increment of 10 years for age, and an increment of 100 μm for SRF height and SFCT

**Fig. 1:**
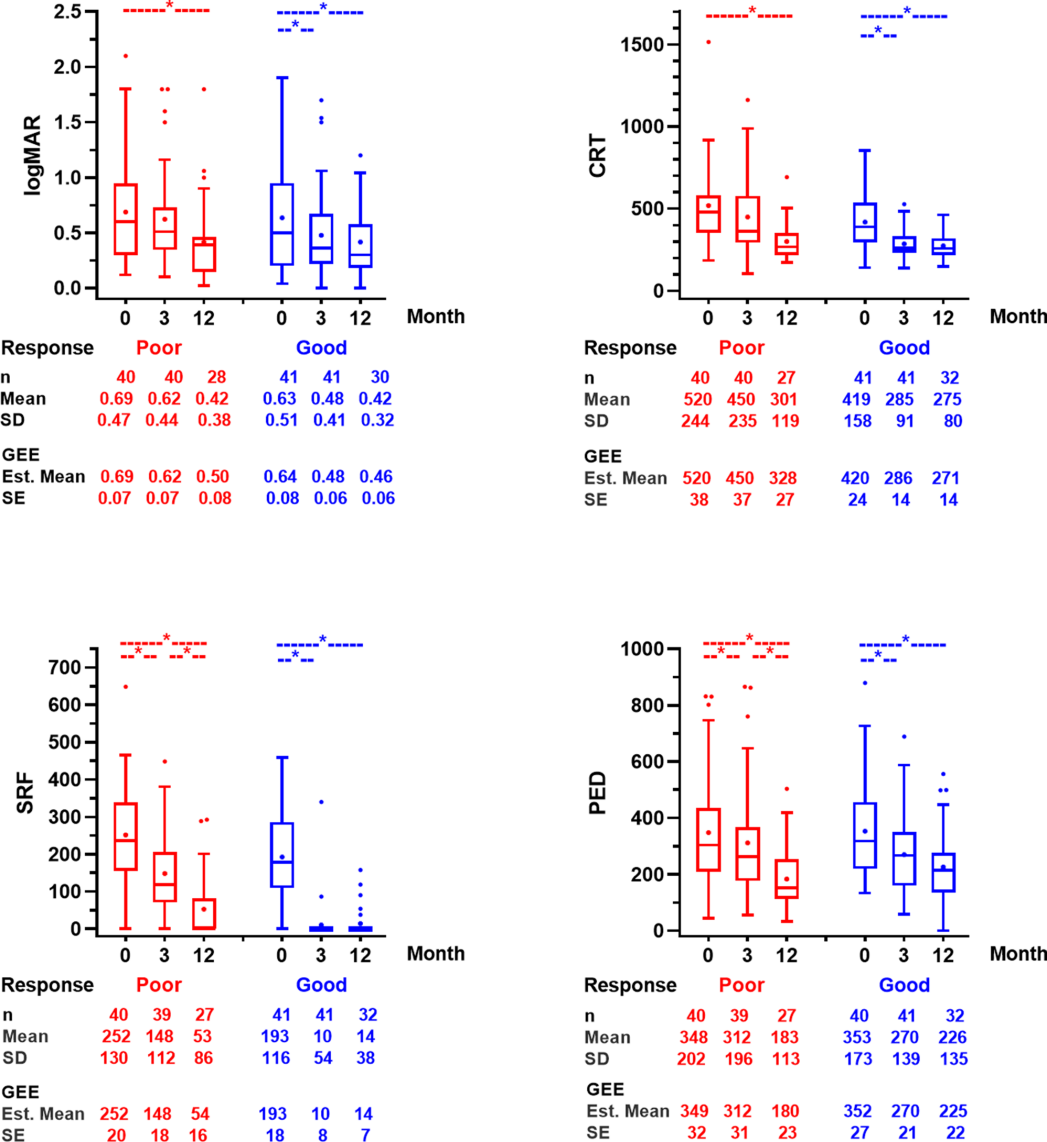
Comparison of VA (logMAR), CRT, SRF height, and PED height between poor response and good response group at baseline (month 0), month3, and month 12

VA, CRT, SRF height, and PED height were assessed within each group over time and between groups at baseline (month 0), month 3, and month 12 (Fig. 1 and Supplementary file 1). At month 12, follow-up data were available for 32 patients in the good response group and 28 in the poor response group.

In the good response group, all parameters improved significantly at month 3 compared with baseline (M0 vs. M3; VA 0.64±0.08 vs. 0.48±0.06 logMAR, *p* < 0.001; CRT 419.5±24.3 vs. 285.5±14.1 μm, *p* < 0.001; SRF height 192.6±18.0 vs. 10.4 ± 8.4 μm, *p* < 0.001; PED height 352.4±26.8 vs. 270.4±21.4 μm, *p* < 0.001) and remained stable without significant changes through month 12.

In the poor response group, VA and CRT showed no significant change at month 3 compared with baseline but achieved significant improvement by month 12 (M0 vs. M12; VA 0.69±0.07 vs. 0.50±0.46 logMAR, *p* = 0.033; CRT 519.7±24.3 vs. 328.4±26.7 μm, *p* < 0.001). SRF and PED heights decreased significantly at both month 3 (M0 vs. M3; SRF height 251.7±20.3 vs. 147.9±17.7 μm, *p* < 0.001; PED height 348.5±31.6 vs. 311.6±30.5 μm, *p* = 0.006) and month 12 (M3 vs. M12; SRF height 147.9±17.7 vs. 53.9±16.2 μm, *p* < 0.001; PED height 311.6±30.5 vs. 180.4±23.2 μm, *p* < 0.001).

Between-group comparisons showed no significant differences in VA or PED height at any time point. CRT was significantly higher in the poor response group at baseline (*p* = 0.027), and month 3 (*p* < 0.001), while SRF height was higher in the poor response group at baseline (*p* = 0.029), month3 (*p* < 0.001), and month 12 (*p* = 0.022).

After the loading phase, 37 of 81 eyes (45.7%) were completely free of IRF and SRF on OCT, all of which were in the good response group. By month 12, 41 of 60 eyes (68.3%) were fluid-free. Of these, 26 eyes (81.2%) were in the good response group, while 15 eyes (53.6%) were in the poor responder group.

In the good response group (*n* = 41), 24 eyes continued bevacizumab on a treat-and-extend regimen (non-switch group), 8 eyes were switched to another anti-VEGF agent, and 9 eyes were lost to follow-up before month 12. In the poor response group (*n* = 40), 24 eyes were switched to other medications (switch group), 2 eyes remained on bevacizumab, 1 eye underwent rescue PDT, 1 eye underwent vitrectomy for breakthrough vitreous hemorrhage, and 12 eyes were lost to follow-up. Excluding the patients who were lost to follow-up, 75% of good response group were continued on bevacizumab monotherapy, while 86% of poor response group were switched to other anti-VEGF. Among the switch group, 18 eyes (75%) were switched to aflibercept, 5 (20.8%) to ranibizumab, and 1 (4.2%) to brolucizumab. The primary reason for switching was persistent or worsening disease activity on OCT.

**Fig. 2:**
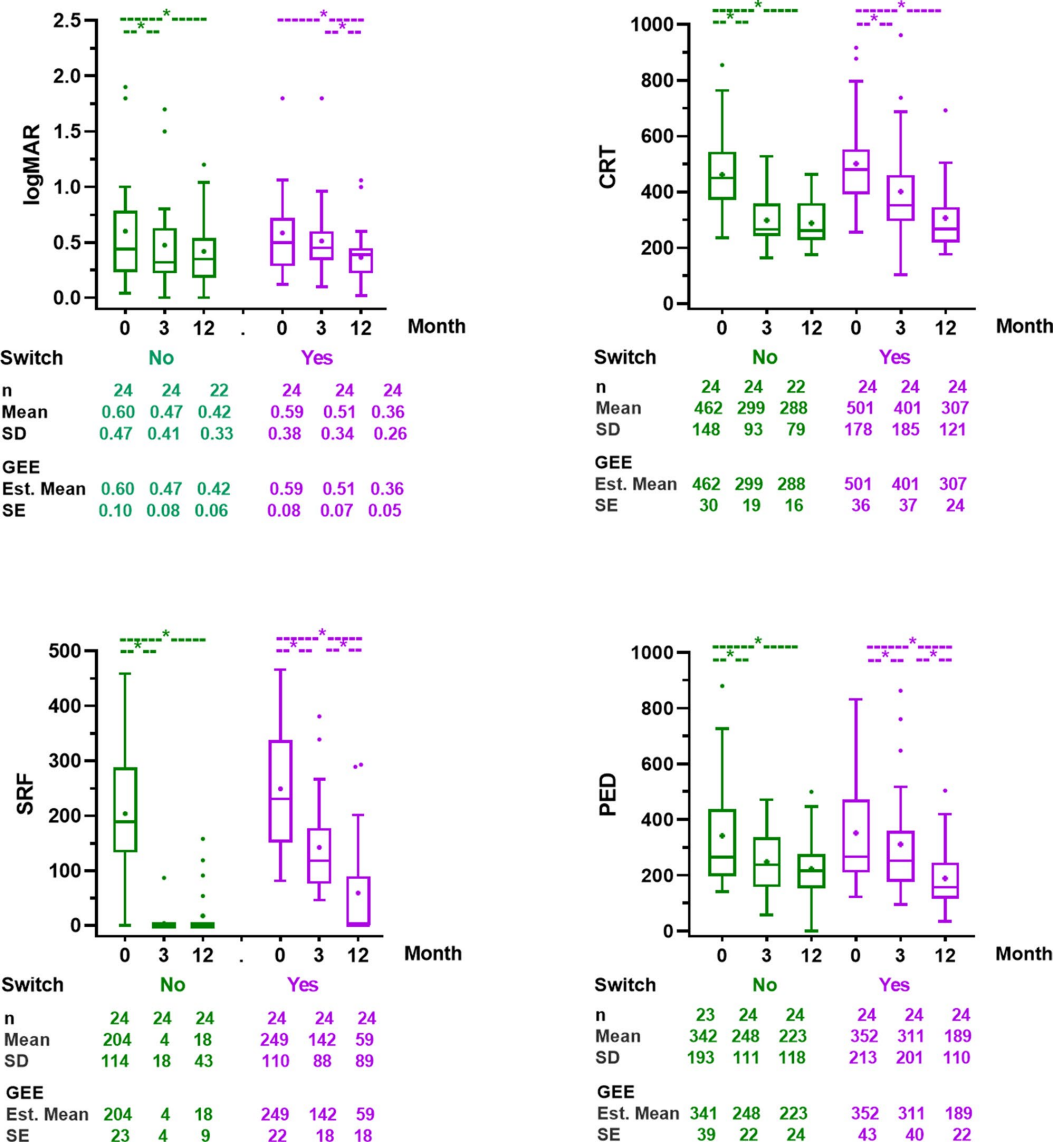
Comparison of VA (logMAR), CRT, SRF height, and PED height between the non-switch group from good responders and the switch group from poor responders at baseline (month 0), month3, and month 12

A subgroup analyses of the non-switch group and the switch group were conducted (Fig. 2). In the non-switch group of good responders (*n* = 24), all parameters showed trends consistent with the overall good response group, with sustained VA and OCT parameters after month 3. In the switch group of poor responders (*n* = 24), VA improved significantly between month 3 and month 12 (0.51±0.07 vs. 0.36±0.02 logMAR, *p* = 0.002). SRF and PED heights were significantly reduced at month 12 compared with month 3 (*p* = 0.001 and *p* = 0.005, respectively). When comparing between groups, only SRF height remained significantly higher in the switch group at month 12 (59.2±17.8 vs. 17.6±8.6 μm *p* = 0.035) (Fig. 2 and Supplementary file 2).

The total number of injections over 12 months was significantly lower in the non-switch group (5.8 ± 2.5 vs. 7.8 ± 3 injections, *p* < 0.001), and the maximum treatment interval in the first year was significant longer (11.7 ± 9 vs. 7.8 ± 3 weeks, *p* = 0.046). Additionally, 11 eyes (45.8%) of good responders continuing on bevacizumab were able to defer injections within the first year.

## Discussion

Due to its favorable cost-effectiveness, bevacizumab is commonly used as a first-line intravitreal therapy for neovascular AMD, including PCV. However, studies specifically evaluating treatment responsiveness and predictive biomarkers of bevacizumab in PCV remain limited, likely due to its off-label status. Our study identified greater baseline SRF height and thinner SFCT as significant independent predictors of a suboptimal anatomical response after three monthly bevacizumab injections. The AUC of 0.79 from ROC analysis incorporating all predictors in the multivariable model indicates good discriminative ability for distinguishing poor responders from good responders. A simplified model using only baseline SRF height and SFCT achieved an AUC of 0.74, reflecting acceptable discrimination. These findings suggest that SRF height and SFCT alone provide reasonably strong predictive information, although the full model offers slightly superior performance. This may assist clinicians in identifying patients who are unlikely to respond optimally to initial bevacizumab therapy, thereby facilitating earlier treatment modification. Additionally, this study demonstrated an approximately 1:1 ratio of good responders to poor responders following a loading phase of three-monthly bevacizumab injections, consistent with previous studies assessing the efficacy of intravitreal bevacizumab and ranibizumab in the treatment of PCV [11, 18]. 

Prior studies have also reported that greater SRF at baseline correlates with persistent fluid and suboptimal anatomical response in neovascular AMD [19, 20]. Conversely, the role of SFCT as a prognostic marker in PCV and neovascular AMD has been inconsistent. Some studies reporting no significant difference between eyes with thin and thick choroid [21–23], whereas another study suggested that a thinner choroid reflects underlying choroidal insufficiency or more advanced disease stages, thereby limiting the treatment effectiveness of anti-VEGF [24]. An ischemic process mediated by VEGF overexpression has been proposed as a key mechanism in the development of polypoidal lesions, similar to typical neovascular AMD, supporting this concept of choroidal compromise in a subset of patients [25]. In contrast, some studies demonstrated an association between greater SFCT with poor response to anti-VEGF therapy [12, 13, 18] It was proposed that increased hydrostatic pressure, choroidal hyperpermeability, and relatively lower intraocular VEGF levels may underlie resistance in eyes with markedly thick choroids [25]. In our cohort, the mean SFCT in poor responders was 214.5 ± 57.2 μm compared with 248.8 ± 77.7 μm in good responders. These values are fall within the medium choroidal thickness range [24]. Thus, our finding of thinner SFCT in poor responders represents relative thinning within a generally medium-thickness choroid, rather than a direct comparison between frankly thin and thick choroid groups. These discrepancies among studies may be partly attributed to ocular and physiologic factors influencing choroidal thickness, including age, gender, axial length, refractive error, and diurnal variations [26, 27]. Moreover SFCT alone is a relatively crude surrogate for choroidal status. Additional choroidal parameters such as the choroidal vascularity index, regional choroidal thickness, choroidal volume, and choroidal contour may provide a more comprehensive characterization of the choroid and its relationship to treatment response, and should be explored in future work [28, 29]. 

In the good response group, VA improvement and CRT reduction achieved at month 3 and were maintained through month 12. These findings are consistent with previous studies demonstrating sustained anatomical and functional improvements in good responders [4, 11]. The mean VA gain of 7 letters and CRT reduction of 134 μm in this group were comparable to results from the ranibizumab monotherapy arm of the EVEREST-II trial which reported a mean BCVA improvement of 5.5 letters and CRT reduction of 109.3 μm at month 24 [7]. These findings supports the use of bevacizumab as an effective, cost-efficient treatment option, especially in resource-limited settings [6]. 

In contrast, the poor response group demonstrated limited VA improvement, CRT, and SRF reduction at month 3, highlighting the limitations of bevacizumab efficacy in certain cases and the need for alternative treatment. 86% of these patients in this group were switched to another anti-VEGF agent, resulting in significant improvements in VA, SRF, and PED height by month 12. Although BCVA at Month 12 was similar between good and poor responders, this likely reflects different time courses of improvement: early gains with subsequent plateau in good responders, and delayed improvement after treatment modification in poor responders. The mean VA gain of 11 letters and CRT reduction of 194.1 μm achieved in this switch group were comparable to the outcomes of aflibercept monotherapy arm in the PLANET study [2]. These findings suggest that in a bevacizumab-first strategy, early identification of non-responders and consideration of switching to a more potent anti-VEGF agent may be beneficial, although this association should be interpreted cautiously given the retrospective design and physician-directed treatment decisions. These findings are consistent with the prior study showing improved outcomes after switching from ranibizumab to aflibercept in unresponsive patients [30]. 

Our subgroup analysis comparing non-switch and switch groups revealed no significant differences in VA, CRT, and PED height at month 12. Only SRF height remained greater in the switch group. These findings demonstrate the feasibility of classifying treatment responsiveness after a three-dose loading phase of bevacizumab. Good responders can often continue bevacizumab with favorable long-term outcomes, fewer injections, and longer treatment interval. Moreover, 11 eyes (45.8%) of good responders who continued bevacizumab injections were able to defer an injection within the first year without recurrence within month 12. However, longer follow-up is required to determine maximum extension interval and recurrence rate.

This study is limited by its retrospective design and relatively small sample size, which may restrict the generalizability of the findings. The absence of ICGA imaging after the initial treatment limited assessment of polyp regression, an important endpoint in PCV management. Nonetheless, this study reflects real-world clinical practice, where repeated use of ICGA is often impractical. In addition, treatment beyond Month 3, including decisions to switch anti-VEGF agents, was based on physician judgment rather than a standardized protocol. This may introduce selection bias and limits our ability to draw causal conclusions regarding the benefit of switching. Future prospective studies with larger cohorts and extended follow-up are warranted to validate predictive markers, investigate long-term outcomes, and refine individualized treatment strategies for PCV patients.

## Conclusions

In conclusion, approximately half of PCV patients achieved a good anatomical response after loading of bevacizumab. Baseline SRF height and thinner SFCT were identified as independent predictors of poor treatment response. Good responders achieved significant early improvements in visual acuity (VA) and central retinal thickness (CRT) that were maintained over 12 months, while poor responders benefited from switching to alternative anti-VEGF agents. Early response assessment after a three-dose loading could guide treatment decisions. Patients who respond well to bevacizumab may continue therapy with fewer injections and longer treatment intervals, whereas early switching in poor responders may be beneficial in improving anatomical outcome.

## Supplementary Information

Below is the link to the electronic supplementary material.

Supplementary Material 1

Supplementary Material 2

## Data Availability

All data generated or analyzed during this study are included in this published article and supplementary information files.
