# Supplementary material for: Predictors of response to bevacizumab monotherapy in polypoidal choroidal vasculopathy: a 12-month retrospective study
Source: Int J Retina Vitreous. 2026 Jan 7;12:23. doi: 10.1186/s40942-025-00795-x (PMC12870351; doi:10.1186/s40942-025-00795-x)
Supplement: Supplementary file 1 — Supplementary Material 1 [file 40942_2025_795_MOESM1_ESM.docx]

|  |  | **Estimated mean (SE)** | |  | **GEE: p-value** | | | **By month** |
| --- | --- | --- | --- | --- | --- | --- | --- | --- |
|  | **Month** | **Poor response** | **Good response** |  | **Group** | **Month** | **Group x Month** | **p-value (group)** |
| VA (logMAR) | 0 | 0.69 (0.07) | 0.64 (0.08) |  | 0.365 | <0.001 | 0.094 | - |
|  | 3 | 0.62 (0.07) | 0.48 (0.06) |  |  |  |  | - |
|  | 12 | 0.50 (0.08) | 0.46 (0.06) |  |  |  |  | - |
|  | 0 vs 3 | 0.455 | <0.001 |  |  |  |  |  |
|  | 0 vs 12 | 0.033 | 0.002 |  |  |  |  |  |
|  | 3 vs 12 | 0.06 | 1.0 |  |  |  |  |  |
| CRT (um) | 0 | 519.7 (38.1) | 419.5 (24.3) |  | <0.001 | <0.001 | 0.033 | 0.03 |
|  | 3 | 450.2 (36.6) | 285.5 (14.1) |  |  |  |  | <0.001 |
|  | 12 | 328.4 (26.7) | 270.9 (13.5) |  |  |  |  | 0.06 |
|  | 0 vs 3 | 0.133 | <0.001 |  |  |  |  |  |
|  | 0 vs 12 | <0.001 | <0.001 |  |  |  |  |  |
|  | 3 vs 12 | 0.007 | 0.397 |  |  |  |  |  |
| SRF height (um) | 0 | 251.7 (20.3) | 192.6 (18.0) |  | <0.001 | <0.001 | <0.001 | 0.03 |
|  | 3 | 147.9 (17.7) | 10.4 (8.4) |  |  |  |  | <0.001 |
|  | 12 | 53.9 (16.2) | 13.8 (6.6) |  |  |  |  | 0.02 |
|  | 0 vs 3 | <0.001 | <0.001 |  |  |  |  |  |
|  | 0 vs 12 | <0.001 | <0.001 |  |  |  |  |  |
|  | 3 vs 12 | <0.001 | 1.0 |  |  |  |  |  |
| PED height (um) | 0 | 384.5 (31.6) | 352.4 (26.8) |  | 0.932 | <0.001 | 0.037 | - |
|  | 3 | 311.6 (30.5) | 270.4 (21.4) |  |  |  |  | - |
|  | 12 | 180.4 (23.2) | 225.3 (22.4) |  |  |  |  | - |
|  | 0 vs 3 | 0.006 | <0.001 |  |  |  |  |  |
|  | 0 vs 12 | <0.001 | <0.001 |  |  |  |  |  |
|  | 3 vs 12 | <0.001 | 0.127 |  |  |  |  |  |

**Supplemental File 1 – Comparison of VA, CRT, SRF height, PED height at month 0, 3, and 12 within and between poor response and good response group**
