# Supplementary material for: Predictors of response to bevacizumab monotherapy in polypoidal choroidal vasculopathy: a 12-month retrospective study
Source: Int J Retina Vitreous. 2026 Jan 7;12:23. doi: 10.1186/s40942-025-00795-x (PMC12870351; doi:10.1186/s40942-025-00795-x)
Supplement: Supplementary file 2 — Supplementary Material 2 [file 40942_2025_795_MOESM2_ESM.docx]

|  |  | **Estimated mean (SE)** | |  | **GEE: p-value** | | | | **By month** | |
| --- | --- | --- | --- | --- | --- | --- | --- | --- | --- | --- |
|  | **Month** | **Non-switch (Good response)** | **Switch**  **(Poor response)** |  | **Group** | **Month** | | **Group x Month** | | **p-value (group)** |
| VA (logMAR) | 0 | 0.60 (0.10) | 0.59 (0.08) |  | 0.914 | <0.001 | | 0.264 | | - |
|  | 3 | 0.47 (0.08) | 0.51 (0.07) |  |  |  | |  | | - |
|  | 12 | 0.42 (0.06) | 0.36 (0.05) |  |  |  | |  | | - |
|  | 0 vs 3 | 0.004 | 0.184 |  |  |  | |  | |  |
|  | 0 vs 12 | 0.013 | <0.001 |  |  |  | |  | |  |
|  | 3 vs 12 | 0.805 | 0.002 |  |  |  | |  | |  |
| CRT (um) | 0 | 461.6 (29.6)) | 501.3 (35.5) |  | 0.08 | <0.001 | | 0.182 | | - |
|  | 3 | 298.5 (18.5) | 401.0 (37.0) |  |  |  | |  | | - |
|  | 12 | 287.9 (15.8) | 307.2 (24.2) |  |  |  | |  | | - |
|  | 0 vs 3 | <0.001 | 0.01 |  |  |  | |  | |  |
|  | 0 vs 12 | <0.001 | <0.001 |  |  |  | |  | |  |
|  | 3 vs 12 | 0.789 | 0.134 |  |  |  | |  | |  |
| SRF height (um) | 0 | 203.9 (22.8) | 248.7 (22.0) |  | <0.001 | <0.001 | | <0.001 | | 0.157 |
|  | 3 | 3.6 (3.5) | 142.2 (17.6) |  |  |  | |  | | <0.001 |
|  | 12 | 17.6 (8.6) | 59.2 (17.8) |  |  |  | |  | | 0.035 |
|  | 0 vs 3 | <0.001 | <0.001 |  |  |  | |  | |  |
|  | 0 vs 12 | <0.001 | <0.001 |  |  |  | |  | |  |
|  | 3 vs 12 | 0.19 | 0.001 |  |  |  | |  | |  |
| PED height (um) | 0 | 340.8 (39.0) | 352.2 (42.7) |  | 0.732 | <0.001 | | 0.039 | | - |
|  | 3 | 248.4 (22.2) | 310.9 (40.1) |  |  |  |  | | | - |
|  | 12 | 222.6 (23.6) | 188.9 (22.0) |  |  |  |  | | | - |
|  | 0 vs 3 | 0.005 | 0.024 |  |  |  |  | |  | |
|  | 0 vs 12 | 0.001 | <0.001 |  |  |  |  | |  | |
|  | 3 vs 12 | 0.627 | 0.005 |  |  |  |  | |  | |

**Supplemental File 2 – Comparison of VA, CRT, SRF height, PED height at month 0, 3, and 12 within and between Non-switch and Switch group**
